# Hospital and Institutional News

**Published:** 1912-09-14

**Authors:** 


### HOSPITAL AND INSTITUTIONAL NEWS.

### THE BIRMINGHAM HOSPITAL CONFERENCE.

The full programme of the British Hospitals Association's Conference in Birmingham, which will be held on Thursday, September 19, and Friday, September 20, is now settled. Thanks to the courtesy of the Lord Mayor of Birmingham, Alderman W. H. Bowater, the meetings will be held in the Council Chamber, where he will open the proceedings with an address of welcome on Thursday morning at ten o'clock. Before lunch, time will be divided between Sir William Collins' eagerly anticipated paper on "Hospitals and the State" and Mr. Edgar Kemp's paper on "The Training and Work of the Almoner." The papers will be followed by a discussion. In the afternoon the Insurance Act will absorb the attention of members, and two papers will be read by Dr. Nathan Raw, M.D., of Liverpool, and by Dr. Basil Rhodes, secretary of the North Staffordshire Infirmary. At five o'clock a reception will be held by the Lord Mayor in the Council House. On Friday one paper only will be read—Mr. Danvers Power's on "Hospital Management considered with Reference to Responsibility," while the afternoon will be given up to visiting the Birmingham hospitals, all of which will be open to the inspection of members of the Association from 2.30 to 4.30., or for those who prefer it Cadbury's Works at Bournville, Elkingtons, the Small Arms Company, or Taunton's bedstead factories can be visited. As was the case at the Manchester Conference last year, arrangements have also been made for those who intend to stay till Saturday to visit such typical Midland hospitals as those at Derby, Leamington, Redditch, Worcester, West Bromwich, and Stoke. The president of the Conference is Mr. J. B. Clarke, J.P., chairman of the board of management of the Birmingham General Hospital, and the hon. local secretary is Mr. Howard J. Collins, of the same institution. The papers which have been arranged for show how alive the Association is to the problems of the day, and it is very important that everyone interested in hospital administration should become a member, not merely for his own sake, but in order that at such times of crisis as new legislation or other causes may produce, the voluntary hospitals of the country generally may have an organ at hand to express, and, if necessary, to enforce, their point of view through a thoroughly representative and responsible body.

### THE END OF OVERCROWDING AT LINCOLN.

The Lincoln Board of Guardians has decided, on the recommendation of the House and Building Joint Committee, to apply to the Local Government Board for power to borrow £20,000 to purchase suitable land on which to build a new infirmary. This is the result of the drastic report on overcrowding which the Board's inspector presented the other day, when the guardians' reluctance to act on it was criticised in these columns. No discussion followed and the Committee's minutes were adopted, only Mr. Noble and Mr. Barlow voting against. The congratulations of the Chairman, Mr. J. Laverack, on "the natural manner" in which a minute involving an expenditure of £20,000 was passed quickly revealed that the silence of the Guardians was not wholly indicative of approval. We have agreed, said Mr. Watkin, to see whether permission be gained; we can decide afterwards whether the money shall be spent. Mr. Laverack, in reply, pointed out that the Board could not be approached in such an off-hand manner, but would want to know how the money was to be spent. This, of course, is the case, but now that the first step has been taken we hope that the end of the overcrowding in the infirmary, which might almost be described as a chronic condition of affairs, is at last in sight.

### THE LOCAL GOVERNMENT BOARD AND INSTITUTIONAL MANAGEMENT.

What purports to be a summary of the new scheme of Poor-Law reform, which is to be embodied in a report from the Departmental Committee of the Local Government Board, appeared in the *Times* of September 11. We are officially informed, however, that no details are available at present, and therefore till we receive them in due course shall refrain from comment. The scheme is understood to aim at simplifying the Poor-Law orders for the management of institutions and at the better classification of themselves and their inmates. It would be strange, indeed, if matter so nearly affecting institutional concerns as the new scheme is likely to do were withheld from the organs of institutional opinion, and its unauthorised appearance in a lay newspaper suggests a leakage that will doubtless be made the subject of inquiry.

### A BROMPTON HOSPITAL VETERAN.

The death on September 10 of Dr. R. E. Thompson removes one who was a familiar figure in medical circles, and especially at the Brompton Hospital for Consumption and Diseases of the Chest, some years ago. Born in 1834, the son of Serjeant Thompson, he was educated at Cambridge and St. George's Hospital; and his successive qualifications were M.B. 1860, M.R.C.P. 1862, M.D. 1863, F.R.C.P. 1868. For some years in the sixties Dr. Thompson was medical registrar at St. George's, but he never reached the staff. In 1869 he became assistant physician to the Brompton Hospital; was physician 1880-94, and consulting physician 1894-1901. He was also assistant physician to the Seamen's Hospital from 1871 to 1873. Dr. Thompson was secretary of the Royal Medical and Chirurgical Society in 1880, and vice-president in 1883. He wrote much on consumption during the years of his professional activity. He was also very fond of music and painting; but of his non-medical career the most interesting part was his journey in 1861 with the late Viscount Milton to the Canadian North-West, at that time an unknown country, where tribes of Indians roamed the prairies in pursuit of vast herds of bison. It was this journey, and that which Lord Milton undertook a year later with the late Dr. Cheadle, which led to the conception and foundation of the Canadian Pacific Railway.

### WHOLE-TIME ARCHITECTS FOR MENTAL HOSPITALS.

To those concerned with mental hospital construction the sixty-sixth Report of the Commissioners in Lunacy cannot fail to be interesting. An average expenditure of about £3,442 on each of the eighty-six schemes dealt with for alterations, additions, and improvements to existing county or borough asylums shows that the architect's department is a busy one, and it is further instructive to note that elaboration of design for mere architectural effect is being discouraged. Evolution in design which may possibly tend to a better solution of the varied and difficult problems in the more successful treatment of patients is not deprecated, but the strictest economy compatible with good workmanship is insisted upon. The temporary arrangement whereby the entire services of an experienced architect were retained for a period of three years is reported to have been most successful, and the Treasury has sanctioned its continuance upon a permanent basis.

### ISLINGTON HOSPITALS LIT BY CANDLES.

On Monday last there was a sudden failure of the electric light in the Islington Borough with the result that considerable trouble was caused to some of the charities situate within its borders; of these only one, we believe, possesses its own electric plant, that being the London Fever Hospital, whose electrical department is a model of what such things should be. The Great Northern Central Hospital in the Holloway Road, which is lighted from the borough mains, was forced to rely for the time being on candles, lamps, and disused gas brackets. The incident should show hospital governors the advisability of having two supplies of light from different sources, in those cases at least in which they cannot afford to manufacture their own illuminant. Separate electric plant is of course the ideal. It is in fact the only safe and sure method in these days of strikes and failures. Nor, when everything is considered, does it come much more expensive than taking the light from the borough mains. The initial cost of the plant is somewhat depressing to the economically minded manager, but it is economy, after all, to secure the best material and to have the surest safeguards against accidents.

### THE COST OF ISOLATION HOSPITALS.

The results of the Local Government Board inquiry into the cost and construction of isolation hospitals have been issued in a report by Dr. H. Franklin Parsons. So many local authorities bring proposals before the Board which are too costly in character that the object of the investigation was to furnish instances of satisfactory hospitals, built at a reasonable cost, which might be referred to as examples. Sanitary authorities and their officers will find much useful information in this report when plans for new isolation hospitals are under consideration. To-day a larger area can be secured than was formerly the case, and improved means of communication enable a single joint hospital, in Dr. Newsholme's opinion, which prefaces the report, to secure important economies over separate institutions. Dr. Parsons, in an interesting chapter on cost, argues that any attempt to estimate the cost of hospitals at so much per bed leads to fallacious comparisons unless other factors, such as size, are taken into account. A small hospital, where these considerations are omitted, will appear to cost more per bed than a large one, as the cost of the administrative buildings and the site cannot be proportionately less. On general grounds of economy, then, Dr. Parsons favours the joint hospital, a careful selection of the site (opposition has commonly led to the acquisition of a larger site than is otherwise necessary), and the employment of a competent architect, and on occasion a reduction of space to 1,872 cubic feet per patient. The second part of the report is the outcome of the visits paid by Dr. Parsons and Mr. Kitchin, the Board's architect, to twenty-four hospitals, which are described, their cost being compared in the light of local influences. In answer to the question of what use have isolation hospitals been in preventing the spread of disease, we are reminded that "the institutional segregation of notified cases cannot realise its potential value unless it is associated with diligent search for unrecognised cases of disease." A useful note is added in one of the appendices comparing the cost of isolation hospitals with that of mental hospitals, in which Dr. Parsons remarks that "land for an asylum seems to be obtainable at a much cheaper rate per acre than it usually is for an isolation hospital"—a fact which drives home the necessity of correcting the cost per bed with other considerations when the cost of isolation hospitals is being compared.

### OPERATIONS AND CHRISTIAN SCIENCE.

The new cottage hospital at Axminster has inaugurated its theatre by surgically treating a Christian Science patient. His restoration to health is understood to have confirmed his belief in the superiority of Eddyism, which, if true, is a striking example of the wonderful way in which human beings can separate belief from practice, as his gratitude to the hospital staff is stated to be extreme.

### THE REFORM OF THE MILK SUPPLY.

The question of the best methods of securing a pure milk supply is again attracting attention, and Dr. Haydn Brown has drawn on his personal acquaintance with dairy habits and trade tricks to put forward various simple safeguards. Among them he emphasises the importance of cows being inspected by veterinary officers who are not local men and who do not practise in the county; he also believes much might be done by publishing in the local papers the Councils' reports on the conditions of the dairies, the cows, the storing and delivery, such reports being made once a quarter. He urges, moreover, that the medical officer of health should act as adviser to the local Council on milk when required, but that his advice should be given only in committee, and that no reports on milk should be included in his own public compilation. Dr. Brown believes that these methods, which are not expensive, would lead good busines men to try for a good report for the sake of the advertisement it would give them. Certainly experience generally proves that reforms in administration, if well thought out, frequently achieve better results than the expensive outlay on new officials which new legislation so often seems alone to produce.

### HOSPITAL GOVERNORS AND MR. LLOYD GEORGE.

The see-saw of medical opposition to the Insurance Act still continues, though the repentant doctors are in a very conspicuous minority. Thus three members of the Newcastle Insurance Committee have declined to obey the call of the British Medical Association to resign their positions on the local committees. But, and it is a striking example of the vigorous opposition of the profession generally, the honorary medical staff of the Macclesfield Infirmary have intimated that their services will probably be terminated under the pledge which they have signed. The result has been that the infirmary governors have unanimously passed a resolution appealing to Mr. Lloyd George to make terms with the medical profession in the interests of the voluntary hospitals and of the medical treatment of the sick poor. We wonder that such resolutions have not been more frequent, for they form a strong background to the action of the medical staffs. Perhaps at next week's Conference at Birmingham those discussing the Insurance Act on the second day's meeting of the British Hospitals Association will raise the question how far boards of management can support the action of medical staffs who follow the line taken by that of Macclesfield Infirmary.

### THE REGISTRATION OF NURSING HOMES.

An Association has now been formed for the protection and registration of nursing homes. The main objects of the Association are described as to ensure that the matron and nurses of a home should hold a certificate of a recognised training school, that the homes should be safeguarded from unjust comment, and that the public should be protected from undesirable homes. Members of the Association will pay a subscription, any surplus which may exist after administration expenses have been paid will be vested in trustees for furthering its objects. The supporters of the scheme include Dr. Mary Scharlieb, Dr. Addison, M.P., Mr. Joseph Francis, chairman of the City of London Lying-in Hospital, and Dr. T. J. Horder. The Association, however, will be well advised to explain somewhat more fully the methods which it proposes to adopt, for it is precisely over these that opinions will differ. Indeed the second and third aims of the Association require very careful explanation, particularly the second, for methods for protecting any institution from unjust comment have seldom been efficacious, since the only adequate course is to secure and preserve internal efficiency and good management, and those which have not already done so are the very homes that will not voluntarily register.

### LIBRARIES WITH LOCKED DOORS.

A correspondent writes to us—*à propos* of the article on medical libraries in a recent issue (The Hospital, for August 17, 1912, p. 517)—to complain that the libraries of the Royal College of Physicians and of the Royal College of Surgeons are both closed throughout the whole of September. He argues that, if it really is necessary to shut up these libraries for a month at a time, at least some arrangement might be made whereby both should not be shut at the same time. There is certainly some force in this argument. Indeed, for our own part we should require some convincing that there is any real need to shut these institutions at all. The staff, no doubt, require holidays; but surely some division of labour could be arranged whereby one member of it is absent at a time and the others between them carry on his work. The hours during which these collections are open are so short that we cannot believe the staff are really overworked. The British Museum, on the one hand, and such institutions as Mudie's, on the other, do not think it necessary to shut up shop for a month in the summer, and we cannot quite see why in the twentieth century the Royal Colleges should do so either.

### A NOTABLE MEDICAL WOMAN.

The death of Dr. Elizabeth Louise Catharine Appel in India removes a woman doctor whose activity and interest in her profession were remarkable, as the following list of appointments held at various times by her may help to show: Medical registrar and assistant anæsthetist at the Royal Free Hospital, house surgeon and lecturer at Clapham Maternity Hospital, demonstrator in anatomy at the London School of Medicine for Women, and at one time physician to the National Anti-Vivisection Hospital—these do not exhaust her appointments. As her lectures showed, she was keenly interested in midwifery, an inspiring teacher, and her "How to Become a Certified Midwife," published by The Scientific Press, 28 Southampton Street, Strand, was a model manual of its class. It retained, indeed, the affection of the public so well that as time passed it had to be brought up to date and revised, a task which was performed by Dr. Victoria E. M. Bennett, herself author of "Lectures to Midwives."

### A GERMAN "BURDETT."

The new edition of Leineweber's "Adressbuch der Krankenpflege und Wohlfahrts-Anstalten Deutschlands" may be classed as the German "Burdett's Hospitals and Charities." It is a volume of nearly 700 closely printed pages and gives the latest and most authentic information concerning the hospitals of the Fatherland. From it we find that there are to-day in Germany 9,054 hospitals with a total of 735,579 beds. Of these 3,258 are general hospitals with a total of 215,908 beds. The special hospitals are classified under different heads thus: children's hospitals, 258 (21,086 beds); gynæcological hospitals, 333 (10,811 beds); eye hospitals, 261 (7,333 beds); orthopædic hospitals (excluding cripple asylums), 104 (4,996 beds); skin hospitals, 78 (2,479 beds); ear, nose, and throat hospitals, 120 (2,690 beds); and hospitals for nervous diseases 195 with 13,501 beds. It must be remembered that many of these are private hospitals of the nature almost of our nursing homes. There are 62 general or special hospitals belonging to approved insurance societies with an average of 160 beds each, and 351 military and naval hospitals. It comes rather as a surprise to find that there are only 281 tuberculosis sanatoriums, homes, and hospitals with a total of 35,533 beds, especially when one remembers that most of the homes are private institutions with only a few beds and a relatively high cost per bed. There is reason to believe that Germany is gradually modifying her sanatorium system and erecting cheaper and less imposing buildings for the care of her consumptives in the shape of forest colonies and camps, which would not come under the heading sanatoria. The Adressbuch shows that 261 hospitals are being built and gives interesting particulars concerning their plans and costs. Regarding the total finances of the hospitals little is said, but an attempt is made to estimate the value of the existing hospitals, which is put at 3 milliards of marks. This is obviously an underestimate, for it takes £200 as the average cost per bed, which is far from being the case. The Adressbuch is well printed and bound and is published at 12s. net.

### COLOURED PATIENTS.

The *South African Medical Record* has some sensible remarks on the subject of the recent agitation in the newest of the Dominions to urge that "black or coloured patients should be nursed only by coloured nurses." This doctrine, which we fancy has to thank for some of its popularity the exaggerated fear of a "black peril," was enunciated in the Provincial Council when the new hospital ordinance (which we hope to discuss in a future issue) was under discussion. As our contemporary justly remarks the demand is on the face of it absurd. If there is any special reason why white nurses should not nurse native patients it is obvious that the nurses would have mooted the question, and that it would not have been left for the lay enthusiasts to point out the undesirability of having a European nurse in a ward which contains native patients. With this view we entirely agree. There is no reason, of course, why native women should not be trained as nurses; as a matter of fact what we have seen of native sick nurses in America, the West Indies, and Java leads us to suppose that they will be every way as capable as white nurses. But nursing, whether in hospital or in private, is not a matter of colour or race, and it is as absurd to contend that a white woman should not nurse a native in the ordinary discharge of her hospital duties as it is to argue that a woman should not nurse a man. We may add that there does not seem to be any likelihood of the new doctrine finding permanent and authoritative popularity at the Cape.

### THE LORD MAYOR'S RETIREMENT.

On Saturday, the 28th inst., the Livery of the various London Guilds meet for a special service at St. Lawrence Jewry. Immediately after the service comes the ceremony of electing a new Lord Mayor in the place of Sir Boor Crosby, M.D. We understand that the senior alderman, Sir David Burnett, will be unanimously chosen. In the evening the Lord Mayor will give a banquet at the Mansion House in honour of the Lord Mayor elect. With this ceremony the present Lord Mayor's official duties do not end. Sir Boor Crosby, of course, continues chief citizen of London until November 9, when his successor will be sworn in. During his year of office Sir Boor Crosby has shown himself worthy of his high office, and the members of the medical profession will join with us in congratulating him upon a Lord Mayoralty which compares excellently with that of any of his predecessors.

### A HOSPITAL SECRETARY'S STORY.

Sir Joseph Pullar, whose death in his eighty-fifth year was announced on Monday last, was a good friend to the hospital and charities of his native town. But he did not confine his philanthropy to Perth nor even to Scotland. It is related that a colonial hospital secretary, visiting Perth, called upon Sir Joseph and claimed an annual subscription for his institution on the score that some of the hospital curtains were "probably dyed by Pullars." Such temerity had its reward in an annual three-figure cheque which we believe reached the hospital regularly for several years. Sir Joseph's main interest outside his well-known business and political activities lay in scientific education. He recognised fully the need for helping struggling students of science, stimulated undoubtedly by the splendid success of his friend Dr. Perkins, to whose discovery of aniline as a dye Perth owes much of its present prosperity. Sir Joseph was one of the Carnegie Fund Trustees and an active and very helpful member of the Technical Education Committee of Scotland. In the hospital world he was connected with the County and City Royal Infirmary and with the Perth Sick Poor Nursing Association. But far more than to either of these two interests he gave his attention to the well-known Hillside Home which he presented to the town (or rather to "the Society for the treatment of chronic disease and the relief of incurables in Perth and Perthshire" of which he was chairman), and which cost close upon £15,000. The home has about ninety beds and nearly £50,000 in invested funds, and Sir Joseph was always its staunchest supporter. He was knighted in 1898, and in the same year gave a public bath house to Perth, a gift which he duplicated some years later.

### A MEDICAL EXAMINATION FOR CHAUFFEURS.

The unfortunate death of a motor-car driver recently, under conditions of a somewhat peculiar nature, has again drawn attention to a point on which The Hospital has expressed itself strongly in the past—the need for proper medical inspection of all motor-car drivers. There is far too great a tendency to hold that anyone is fitted to drive a motor vehicle. Indeed it is sometimes even regarded as justifiable to appoint as chauffeur a man who is unable to do other manual work by reason of some bodily defect. We know a case of a hospital porter who was unable to continue his work and for whom a situation was found as driver of the hospital motor ambulance. It was only when a foreign medical man visited the hospital and saw that the motor driver was obviously unwell that it was discovered that the man suffered from advanced heart disease. We can easily imagine a similar case in which an employer acts with the best and most charitable intentions. But motor driving does not concern merely the chauffeur and the owner of the car: if it did we should not venture to say anything further on the subject, beyond pointing out the obvious danger of catastrophe in cases where the chauffeur suffers from heart disease. The sudden illness or incapacity of a driver in the street may, however, cause disaster to others who are not in the least concerned in allowing such a man to drive a car. In the interests of public safety, therefore, it is desirable that those who are allowed licences to drive cars should be medically inspected. We should make no distinction whatever between the driver of a public or of a private vehicle, and we should most certainly include women drivers. The medical examination should be made by the applicant's own medical attendant or by an official specially appointed, and there is really no reason why such a public safeguard should not be instituted as soon as possible.

### ITALIAN HONOURS FOR ENGLISH SOLDIERS.

Decorations for services rendered in times of emergency by the rank and file, whether of the military or civilians, are apt to be delayed, but should be none the less welcome on that account. It is interesting, therefore, to record that the King has approved the acceptance and wearing of the silver medal of the Italian Red Cross Committee by certain non-commissioned officers of the Royal Army Medical Corps for services rendered by them during the earthquake in Sicily and Calabria on December 28, 1908. The names of those thus honoured are: Corporal W. Smith, Corporal H. E. Barton, and Lance-Sergeant J. A. Chipperfield.

### "THE COMET OF A SEASON."

The question of standard bread has still a surviving interest from the nine days' wonder which a certain section of the press engineered within living memory. Thus Dr. W. A. Bond, Medical Officer of Health for Holborn, urges in his annual report the importance of fixing a standard for the nutritive value of what is sold as bread. He urges also the risk attending the chemical bleaching of flour, and deprecates as highly dangerous the addition of "improvers," as the indiscriminating use of hydrofluoric acid and phosphorus pentachloride are falsely called. The fact is adulteration assumes the name of "improvement," "addition," or "preservative" nowadays, and the careful warnings of medical officers and others are almost powerless to do more than record the evil effects of adulteration in the many forms in which it disguises itself.

### A MEDICAL TRIBUTE TO A SPECIAL HOSPITAL.

It is extremely gratifying to come across such a graceful tribute of appreciation to hospital work as that which Dr. Niven, the Medical Officer of Health for Manchester, pays in his annual report, which was submitted to the City Council a few days ago. "The work done under the notification of ophthalmia neonatorum," he says, "continues to be excellent, thanks partly to the supervision of Dr. Barbara Cunningham, and partly to the excellence of the nurses whom we owe to the public spirit of the Royal Eye Hospital. . . The department of public health is able to record a successful year owing to the manner in which the staff of the Royal Eye Hospital has hitherto helped us to utilise the special skill and knowledge acquired at that institution." And again: "It is hoped that the splendid results obtained and the gratitude earned may compensate them in part for the time taken up and the labour expended." The staff of the hospital cannot fail to appreciate such references as those, which should do much to confirm the support of the city.

### THE PRELIMINARY TRAINING SCHOOL MOVEMENT.

An important step is about to be taken at Queen Charlotte's Hospital in the establishment of a preliminary training school in connection with its large training school for midwives and monthly nurses. In brief, the committee of management have made arrangements whereby those candidates who desire to do so may undergo one month's preliminary training under the supervision of a sister-in-charge before entering upon the ordinary training in the wards. During this month they will receive such preparation and instruction as will fit them to carry out their ward duties, including lectures in elementary anatomy and physiology, instruction in sick-room cookery, and such details of practical nursing as can be taught before actual attendance on patients is undertaken. The knowledge thus acquired will enable the candidates to take to the work more easily and to create less friction when admitted to the wards of the hospital, and this preliminary school should be of great value to the large number of applicants. One by one the various general and special hospitals are finding out the practical value of the preliminary training school.

### COMMITTEE CHANGES AT THE LONDON HOSPITAL.

Resignations and institutional appointments were announced at the quarterly court of governors of the London Hospital which was held last week.

The Hon. Edward Coke and the Rev. Canon Masterman have been added to the house committee, and to the general regret Mr. Raphael has resigned his position as chairman of the Herman de Stern Convalescent Home at Felixstowe. Since the hospital's convalescent home business comes now under the care of the Marie Celeste Society, the Society is to be asked to superintend the work. The chief feature of interest during the quarter has been, of course, the opening of the pupil probationers' preliminary training school at Tredegar House, which was carefully described in a special article in The Hospital at the time of Queen Alexandra's visit.

### DECORATION FOR A MEDICAL MISSIONARY.

Mr. Robert John Gordon, of the Irish Presbyterian Mission, has received the authority of the King to wear the insignia of the Third Class of the Third Grade of the Order of the Double Dragon. This decoration has been conferred on Mr. Gordon by the Emperor of China in recognition of valuable services rendered by him as plague prevention medical officer at Hulan, Heilung-chiang. The one side of missionary work which still needs the most active encouragement is the medical, and such decorations should help to emphasise the opportunities which medicine alone can give to the missionary.

### A PATIENT'S GRATITUDE AFTER TWELVE YEARS.

A striking instance of a patient's gratitude to Addenbrooke's Hospital, Cambridge, has come to hand from Mr. R. J. Coles, secretary-superintendent. Having occasion to visit the porter's lodge at the hospital a day or two ago, and not finding the porter there at the moment as he was just taking a case to the casualty department, Mr. Coles was returning to his office, and, as he passed through the entrance hall, observed a working man standing at the door. On being asked if anything could be done for him, he stated that he had some money for the hospital. Asked into the secretary's office the visitor then produced ten pounds in gold, saying he wished to give this to the hospital. He added: "My name and address do not matter. I was a patient in the Griffith Ward twelve years ago suffering from typhoid fever, and I want you to accept this donation as a slight token of my gratitude for the treatment I then received, and which I shall never forget. I have been saving up ever since to enable me to express my gratitude in a practical way, and I have cycled many miles this morning to bring you my small gift." Needless to say, he was heartily thanked for his handsome contribution, and assured that the committee would greatly appreciate this practical expression of gratitude after so many years.

### COMMON-SENSE COOKERY.

The Society of Medical Officers of Health is to be congratulated upon the success which has attended its dinner given to the London press representatives to demonstrate the palatability and cheapness of common-sense cookery. We have hitherto had far too much attention paid to the fads of the vegetarian enthusiast, who has harped on the fact that meat cookery is expensive. The fourteen-course dinner which the pressmen sampled on Friday, the 6th inst., clearly showed that a wholesome and appetising "mixed" menu could be provided at a cost of less than twopence per head. We trust that the care committees who have charge of the feeding centres attached to the London County Council schools will pay some attention to the recipes and introduce some of them into the schools. At present, we have reason to think, there is an unfortunate sameness in the food supplied to school children. There are, of course, some excellent exceptions (notably at the cooking centres, where the pupils prepare the meals themselves), but on the whole there is not much variety. The dinner provided by the Society of Medical Officers of Health was excellent, both from the point of view of variety and from that of taste. Perhaps the only justifiable objection to it was the use of paprika sauce. Good paprika is relatively expensive, and if the condiment were to become popular in this country it is possible that the powdered beans would be heavily adulterated with ochre. The poor man (or woman) should stick to some cheaper flavouring, such as turmeric or curry powder. We are glad to notice that the Society intends to educate the public further, and is proceeding to organise a definite campaign.

### NAVAL DISPENSERS' DESIRE FOR REFORM.

Application has been made by the dispensers in H.M. Naval Service for recognition by the appellation pharmacist in place of dispenser as at present. In view of the action taken of late by other public bodies, this step may well be sanctioned by the authorities. The Naval Service is, perhaps, the best Service for pharmacists as far as proper recognition of qualification goes, but leaves much to be desired in the matter of a recognised status. For instance, the Naval dispenser holds no commission, neither is he a warrant officer. He can terminate his engagement by three months' notice. Both in this Service and in the Army a thorough overhauling of the conditions under which the compounding of medicine is carried on is necessary. At present a pharmacist who desires to be an Army compounder must be prepared to enlist as a private, and, if selected for compounding duties, can rise only to be a sergeant and receive about 5s. 2d. per diem. Almost every Continental Power has a Pharmaceutical Service, in which commissions as lieutenant, rising to higher grades, are granted.

### TO OUR READERS.

Contributions are specially invited from any of our readers to these columns. They should deal with topical subjects and news. They must be authenticated for the information of the Editor only. The minimum payment if published is 5s. There is no hard-and-fast rule as to space, but notes of about twenty lines in length are preferred.

